# Resistance training improves mobility disability in community-dwelling older adults: a meta-analysis

**DOI:** 10.1093/geroni/igab046.3258

**Published:** 2021-12-17

**Authors:** Christina Prevett, Kevin Moncion, Stuart Phillips, Julie Richardson, Ada Tang

**Affiliations:** 1 McMaster University, Concord, North Carolina, United States; 2 McMaster University, McMaster University, Ontario, Canada

## Abstract

Mobility disability is the impairment in function that affects the performance of daily tasks due to declines in physical function. Exercise interventions, particular resistance training, may have a positive impact on mobility disability, but the evidence for the effects of resistance training in older adults with mobility disability has not been previously systematically reviewed. This study was a systematic review of evidence related to resistance training on physical function for adults over 65 years of age with mobility disability. Four databases (PEDro, MedLine, Ovid, Web of Science) were searched from inception to February 2, 2021 for randomized controlled trials. Twenty-four articles from 22 studies (3,656 participants) were included in the review. Mean participant age ranged from 63-87 years and exercise interventions ranged from 10 weeks to 12 months in duration. Greater changes in 6-minute Walk Test (6MWT) distance (n=638, p<0.0001; mean difference (MD) 16.1 metres; 95%CI 12.3-19.9), lower extremity strength (n=785, p<0.0001; standard MD 2.01; 95%CI 1.27-2.75) and usual gait speed (n=2,106, p<0.001; MD 0.05 metres/second, 95%CI 0.03-0.07) were seen with resistance training as compared to control. These results were maintained if resistance training was a sole intervention or a component of a multi-component program. Sensitivity analysis based on risk of bias concerns did not change results. This review demonstrates that resistance training improves walking capacity, strength and walking speed in community-dwelling older adults and may facilitate aging in place. Since improvements in strength and gait speed contribute to independence, our results indicate highly beneficial outcomes for older persons.

